# Next-Gen Point-of-Care Tool for Ultra-Sensitive Detection of Urinary Spermine for Prostate Cancer Diagnosis

**DOI:** 10.1021/acssensors.4c03250

**Published:** 2025-04-11

**Authors:** Parisa Dehghani, Mostafa Salehirozveh, Ataollah Tajabadi, Chi Chung Yeung, Michael Lam, Hing Y. Leung, Vellaisamy A. L. Roy

**Affiliations:** † James Watt School of Engineering, University of Glasgow, Glasgow G12 8QQ, U.K.; ‡ Department of Physics and Astronomy, University of Bologna, 40126 Bologna, Italy; § School of Science and Technology, Hong Kong Metropolitan University, Ho Man Tin, Kowloon 999077, Hong Kong; ∥ Department of Chemistry, City University of Hong Kong, Kowloon 999077, Hong Kong; ⊥ Cancer Research UK Scotland Institute, Glasgow, ; School of Cancer Sciences, MVLS, University of Glasgow, Glasgow G12 8QQ, U.K.

**Keywords:** molecular-imprinted polymer, spermine, extended-gate field effect transistor, electrochemical technique, prostate cancer

## Abstract

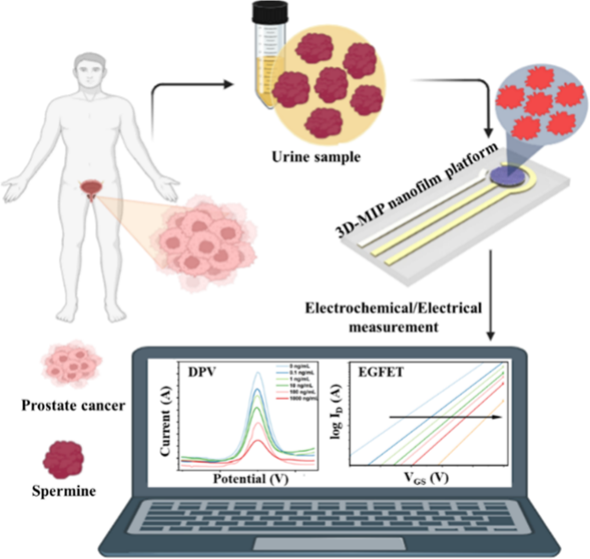

Prostate cancer (PCa), the second most common cancer in men, demands effective early detection strategies. Elevated spermine levels in the prostate tissue contrast with decreased urinary concentrations in PCa patients. Here, we present a novel sensing platform combining differential pulse voltammetry and an extended gate field-effect transistor (EGFET) with a molecularly imprinted polymer|molecular imprinting (MIP) nanofilm for selective and sensitive spermine detection. Key advancements include successfully constructing and characterizing a pseudoreference electrode and a precisely engineered analyte binding interface. The Ag/AgCl pseudoreference electrode exhibited high reliability and reproducibility, optimized to enhance conductivity and minimize interference noises. Electrochemical analysis confirmed successful MIP modification, creating a precise 3D-imprinted binding interface. The platform accurately quantified spermine in artificial urine across concentrations from 0.1 to 1000 ng/mL, achieving a detection limit of 1.23 ng/mL. High selectivity was demonstrated against competing polyamines such as spermidine and histamine. Analysis of electrical properties indicated that spermine binding induced changes in surface potential, altering the metal-oxide-semiconductor field-effect transistor threshold voltage and validating the system’s sensitivity. The system’s superior performance was confirmed with a high imprinting factor (IF ≈ 4.1) and sensitivity 10 times higher compared to nonimprinted polymers. Hill–Langmuir analysis confirmed a strong binding affinity to spermine. Clinical validation using human urine samples from PCa diagnostic evaluations demonstrated high consistency with liquid chromatography mass spectrometry, exhibiting an excellent linear correlation (*R*^2^ = 0.97) without statistically significant differences (*p*-value <0.0001). This study introduces a robust, miniaturized, and cost-effective EGFET-based sensor for spermine detection, offering substantial potential for clinical diagnostics and PCa biomarker monitoring.

Prostate cancer (PCa), the second most prevalent cancer among men, has raised significant global concern. Early detection of PCa not only improves patients’ life expectancy and overall quality of life but also has the potential to reduce the financial burden associated with treatment for both patients and government entities.^1,2^ Early detection strategies using a prostate-specific antigen (PSA) as a PCa-specific biomarker have already been widely implemented, leading to increased success in early PCa diagnosis and a stage migration phenomenon. This means that indolent cancers are being more frequently detected, while metastatic cancers are being detected less frequently in the USA and Europe.^3^ Conversely, limited dissemination of early detection strategies and recommendations that discourage PSA screening for PCa detection might explain why PCa incidence has been lower in some geographical areas and has stabilized or decreased in high-income countries in recent years.^4^ A European study on PCa screening with PSA showed that the screening of 570 men and the treatment of 18 diagnosed patients can prevent one PCa death over a 16 year follow-up period.^5^ However, the use of PSA for the screening, diagnosis, and treatment of indolent PCa results in unnecessary biopsy procedures and treatment. This is due to its general lack of specificity, leading to over 75% of prostate biopsies yielding negative outcomes in the last two decades.^6^ More specific PCa diagnostic tools available today include blood tests (the Prostate Health Index, the 4-kallikrein panel),^7,8^ urine tests (PCA3, SelectMDx),^9,10^ and imaging tests (multiparametric MRI)^11^ allowing for more accurate diagnosis of significant PCa and reducing unnecessary biopsies.^12,13^

The human prostate tissue contains a high level of polyamines, which are involved in the growth and proliferation of prostatic glandular epithelial cells. Studies have revealed that proliferating PCa cells produce higher amounts of polyamines, such as putrescine and spermidine.^5,14^ In benign prostate tissues with large luminal volumes, spermine is predominantly found in the prostate epithelium and plays a role in secretory functions.^14^ However, in the PCa tissue, spermine levels are lower due to poor cellular differentiation, changes in the cellular architecture, and reduced luminal volumes.^5,15^ Pilot study results showed that a lowering of urinary spermine level without a prior prostatic massage has a significant correlation with PCa.^15^ A growing body of evidence indicates that fluctuations in spermine levels, particularly lower concentrations in urine, are strongly associated with various cancers, including PCa. In a cohort of 600 men, reductions in urinary spermine correlated with 3 and 3.5-fold increases in the risks of PCa and high-grade PCa, respectively. Furthermore, incorporation of spermine into a risk score alongside PSA, prostate volume, and digital rectal examination improved diagnostic accuracy (AUC of 0.78 for PCa and 0.82 for high-grade PCa). Additionally, spermine levels in PCa patients can be 7–34 times lower than in healthy individuals and 5–13 times lower than in those with benign prostatic hyperplasia.^5,16^ On the other hand, elevated level of urinary spermine was detected in patients with localized malignant tumors, including lung and liver.^17,18^

A range of techniques have been used to detect spermine in aqueous samples. They include optical,^19−28^ electrochemical,^29−34^ opto-electrochemical,^35^ absorption spectroscopy,^20^ high-performance liquid chromatography,^36^ liquid–liquid phase extraction,^37^ capillary electrophoresis, and mass spectrometry.^38^ Numerous challenges are intrinsic to conventional procedures, encompassing issues such as tedious sample preparation requirements, high costs, protocol intricacy, and the demand for specialized expertise.^39^ These challenges collectively underscore the impetus for exploring alternative methodologies. Electrochemical techniques, owing to their high sensitivity, low cost, and exceptional selectivity, have brought about a revolutionary transformation in analytical detection when juxtaposed with conventional methods. Leveraging its inherent selectivity and strong affinity for specific target molecules, the molecularly imprinted polymer|molecular imprinting (MIP) technique has emerged as a consequential advancement, enabling the electrochemical determination of a diverse range of analytes.^40^ The electrochemical sensing method, when integrated with MIP, shows promise in surpassing conventional techniques for cancer detection. MIPs are synthetic materials designed with artificial molecular recognition properties that mimic natural biological receptors such as antibodies, enzyme active sites, and aptamers.^41^ MIPs fabricated using the target analyte as a molecular template are good analyte-responsive components for sensors owing to their ability to withstand interference from complex sample matrices.^42,43^ MIPs possess unique properties such as mechanical robustness, chemical stability, high affinity to templates, and cost-effectiveness.^44^ By using molecularly imprinted electrochemical sensors, the advantages of both electrochemical methods and MIP can be harnessed.^45,46^

In recent years, the realm of ion-sensitive field-effect transistors (ISFETs) has witnessed burgeoning attention because of their rapid response, elevated sensitivity, minimal energy demands, the capability of amalgamating readouts and circuitry on a singular chip, miniaturization potential, and affordability.^47,48^ The operational principle of ISFETs is based on the correlation between the threshold voltage of a metal-oxide-semiconductor field-effect transistor (MOSFET) and the gate’s ability to modulate, governed by the electric charge of an ion-responsive membrane. Variations in the electrical properties may originate from a chemical reaction within the medium atop the gate. Thus, by immersion of the gate in a solution, the ISFET acquires its distinctive current–voltage characteristics via surface chemical interactions. Nonetheless, within the avant-garde domain of complementary metal–oxide–semiconductor (CMOS) technology, the gate stack emerges as a critical element in the fabrication process, characterized by rigorous standardization, which poses challenges for modification or functional integration for sensing objectives.^49^ To circumvent this issue, a novel approach has been proposed in the guise of extended-gate FETs [extended gate field-effect transistor (EGFET)], specifically conceived to fulfill sensing requisites.^48,50,51^ In this particular sensor design, a traditional MOSFET is employed as the primary transducer, while the detection module is realized through a specialized functional coating applied to an extended gate as an external electrode or a metallic layer connected to the MOSFET gate. The architectural delineation of the functional layers from the integrated transducing unit in EGFET systems confers several advantages, including augmented stability, diminished drift, and attenuated sensitivity to thermal fluctuations.^52^ To the best of our knowledge, no research groups have endeavored to design a spermine MIP sensing platform utilizing FET devices.

Meanwhile, the application of microfabrication techniques and technology has gained prominence in the manufacturing of portable sensing platforms, owing to their inherent advantages such as process simplicity, high efficiency, and cost-effectiveness. However, commercial Ag/AgCl electrodes are unsuitable for integration into microelectrochemical systems due to their macro-scale design. Furthermore, their short lifetime and poor stability are other challenges that pseudoreference electrode miniaturization is facing. Consequently, numerous researchers have focused on miniaturizing and integrating pseudoreference electrodes with other platforms, particularly for biomedical applications. Notably, there has been a rapid growth in studies during the early years of this century, focusing on the miniaturization of Ag/AgCl pseudoreference electrodes. Fabricating a thin layer of the pseudoreference electrode is a critical aspect of its production. Such an electrode typically consists of a chlorinated silver electrode that directly interfaces with the measuring solution. Microfabrication techniques offer an effective solution to address this challenge, leading to the widespread adoption of this approach. Various methods have been developed for the fabrication of microelectrodes, including screen printing, physical (PVD) and chemical vapor deposition, and electrochemical deposition.^53−57^

In this study, we ventured into the realm of a cutting-edge transparent glassy sensing platform featuring an exceptionally stable and reproducible reference Ag/AgCl electrode. This transparent sensing platform has showcased its capability to enhance the effectiveness of MIP methodology. The novel strategy employs MIP within an electrochemical context, leveraging the exceptional host–guest molecular binding characteristics between spermine and the molecularly imprinted binding cavities engineered at the surface of the electrode. This approach is further enriched by the use of electrochemical differential pulse voltammetry (DPV) employing potential pulses. The achieved limit of detection (LOD) for spermine is 1.32 ng/mL, demonstrating the high sensitivity of the electrochemical sensing platform. Furthermore, the platform exhibits exceptional selectivity for spermine (SPM) even in the presence of spermidine, another polyamine with similar characteristics and histamine.

To implement point-of-care (POC) technology on the 3D-MIP-modified glass sensing platform, we employed a MOSFET to detect SPM for the first time. We present the first EGFET that achieves both high sensitivity and selectivity for SPM detection. This choice enables real-time, rapid, and label-free electrochemical detection of the SPM, providing a smart and portable sensing platform for PCa detection. This configuration yields a compact yet powerful electronic sensor. The transparent glassy sensing platform developed in this project seamlessly integrates with a wide array of external devices, rendering it exceptionally suitable for portable laboratory applications. This platform features an integrated highly stable Ag/AgCl pseudoreference electrode, rendering it an exceptionally promising tool for the detection of a wide range of biomolecules.

## Results and Discussion

### Pseudoreference Electrode Characterization

**Figure 1:**
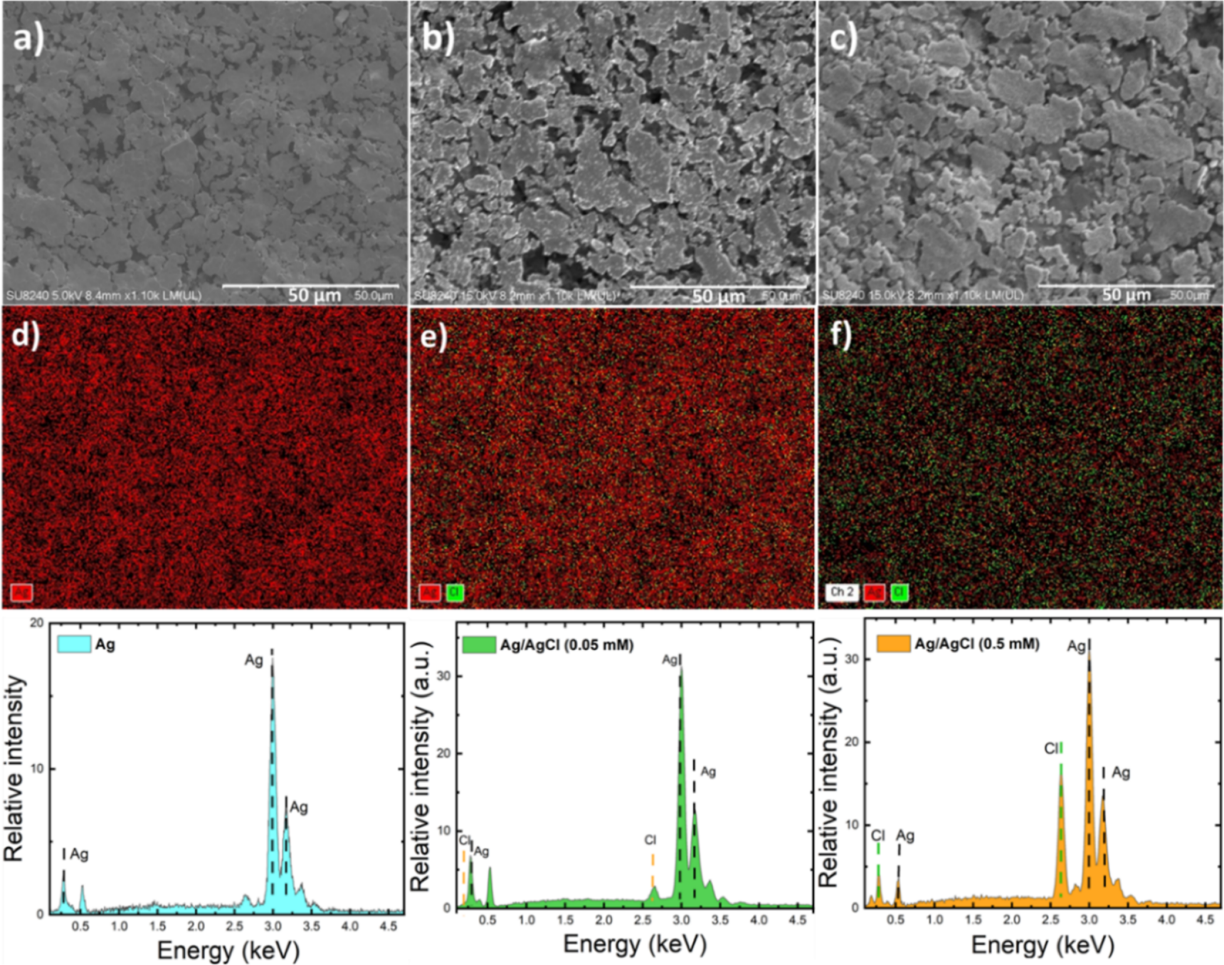
SEM images to study the morphology of the silver electrode before (a) and after exposure to 0.05 mM (b), and 0.5 mM (c) FeCl_3_. The EXD analysis of the silver layer before (d) and after the chlorination process with two different concentrations of FeCl_3_ solution 0.05 mM (e) and 0.5 mM (f) depicts the proper signals of Ag and Cl.

The morphology and performance of the Ag/AgCl electrodes were characterized using SEM, EDX, and CV. As depicted in Figure 1, the surface of the Ag pseudoreference electrode exhibits large silver grains in the range of 1–20 μm before chlorination (Figure 1a). Following exposure to two different concentrations of FeCl_3_ solution, namely, 0.05 and 0.5 mM, the size of the surface granular structure diminished after the formation of the AgCl layer. This is apparent in Figure 1b,c. Increasing the FeCl_3_ concentration led to a larger grain size, enhancing the connection between grains and thus improving the conductivity of the AgCl layer. In addition, the increased grain size can lower the number of pores in the AgCl layer, leading to a reduction in interference noise caused by electrolyte seeping into the underlying Ag layer.^56,58,59^

The presence of the AgCl layer is unmistakably confirmed by EDX through the appearance of chlorine peaks (Figure 1e,f), in addition to the silver peaks, while the silver electrode exhibited only Ag peaks (Figure 1d).^53,60^ The electrode’s performance was assessed through electrochemical characterization using CV in a readout solution. Initially, the performance was evaluated by varying the scan rate and comparing the results to those obtained using a commercial Ag/AgCl electrode. Figure S1a reveals that there is no significant difference between the curves obtained from the fabricated pseudoreference electrode and the commercial one. With the reliability of the pseudoreference electrode confirmed, the repeatability of the electrode was investigated by comparing the CV curves from three different electrodes. As depicted in Figure S1b, the fabricated pseudoreference electrode showed high reproducibility, as there were no significant shifts or differences observed among the three electrodes.

The pseudoreference electrode was successfully fabricated and thoroughly characterized. Moreover, it has showcased its potential to substitute for electrodes. This advancement provides a more economically efficient option for electrochemical analyses as well as significantly contributing to the acceleration of miniaturization.

### Development of the Binding Surface

**Figure 2:**
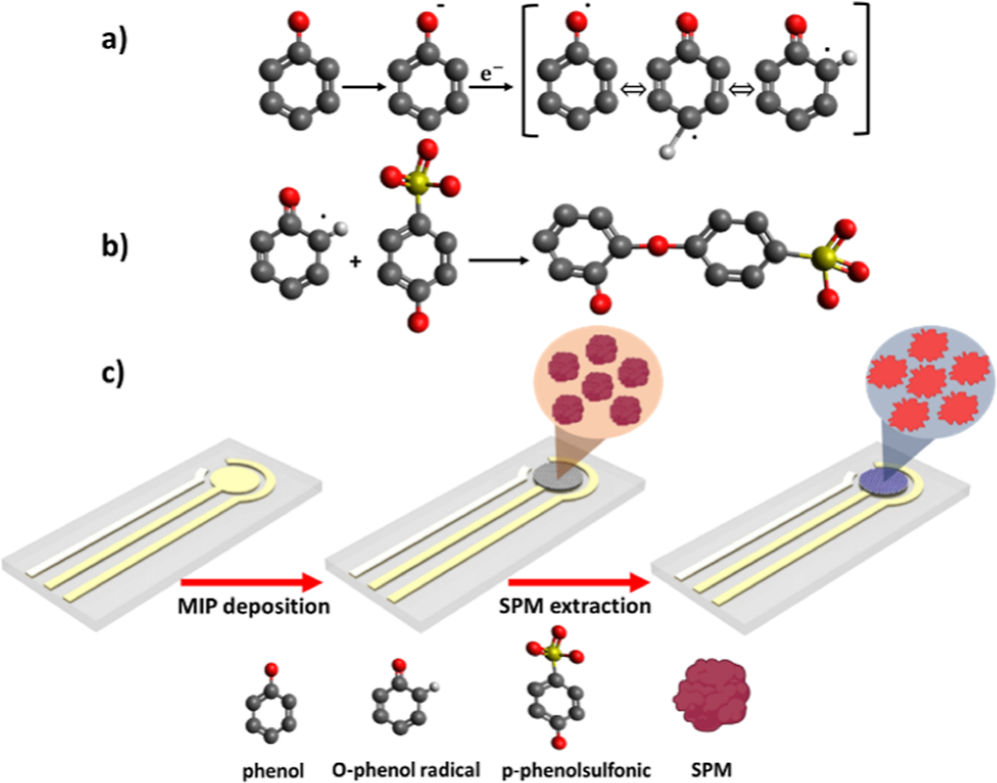
Schematic chemical structure of three distinct isomeric of phenol radicals (a) and interaction between o-phenol radical and p-phenol sulfonic during the electropolymerization (b) and MIP modification (c). After the extraction of the target molecules, the MIP nanofilm records the size, shape, and functional group of SPM molecules.

In the electropolymerization process, phenol underwent deprotonation in an aqueous media. As depicted in Figure 2a, under the influence of an electric field, phenate anions were transformed into phenol radicals at the anode. During the electropolymerization, p-phenol sulfonic acid underwent polymerization at the ortho position of the phenol radical, thereby facilitating the formation of a dimer structure which was primarily responsible for the formation of the observed side chain in electropolymerization processes (Figure 2b).^61−63^ Here, p-phenol sulfonic acid was driven by its ability to form stable noncovalent interactions such as hydrogen bonding and electrostatic interactions with SPM. During the polymerization process, these interactions help create a cavity complementary to SPM’s size, shape, and chemical functionality. After polymerization, the SPM is removed, leaving behind recognition sites that facilitate the highly selective rebinding of SPM. The inclusion of phenol as a cross-linker further stabilizes the polymer matrix, enhancing both the structural integrity of the imprinted cavities and the overall specificity of the MIP sensor.

### Surface Characterization of 3D MIP-Modified Layer on Electrode Surface

Some characterizations like AFM, SEM, EDX, XRD, and FTIR were carried out to characterize the surface of the MIP and the unmodified nonimprinted polymer (NIP) electrodes. These include a series of comprehensive comparisons before and after polymerization, extraction, and the subsequent introduction of SPM as the target analyte.

As depicted in the AFM images (Figure S2a), the thickness and root-mean-square roughness (RMS) increased after the deposition of the MIP nanofilm (Figure S2b). However, the RMS enhancement was significantly more pronounced after the extraction process (Figure S2c), confirming the successful removal of the templates from the polymeric solid-phase scaffold. The introduction of SPM induced a resurgence in surface roughness, a visual testament to the specific interaction between the polymeric scaffold and the target biomolecule. It is evident from Figure S2d that after introducing SPM, the RMS decreased due to the occupation of the molecularly imprinted binding sites.^47,64,65^ As illustrated in Figure S2e,f, there are no significant differences in the thickness and RMS of the NIP nanofilm before and after the extraction step (summarized in Table S1). More information is provided in the Supporting Information.

**Figure 3:**
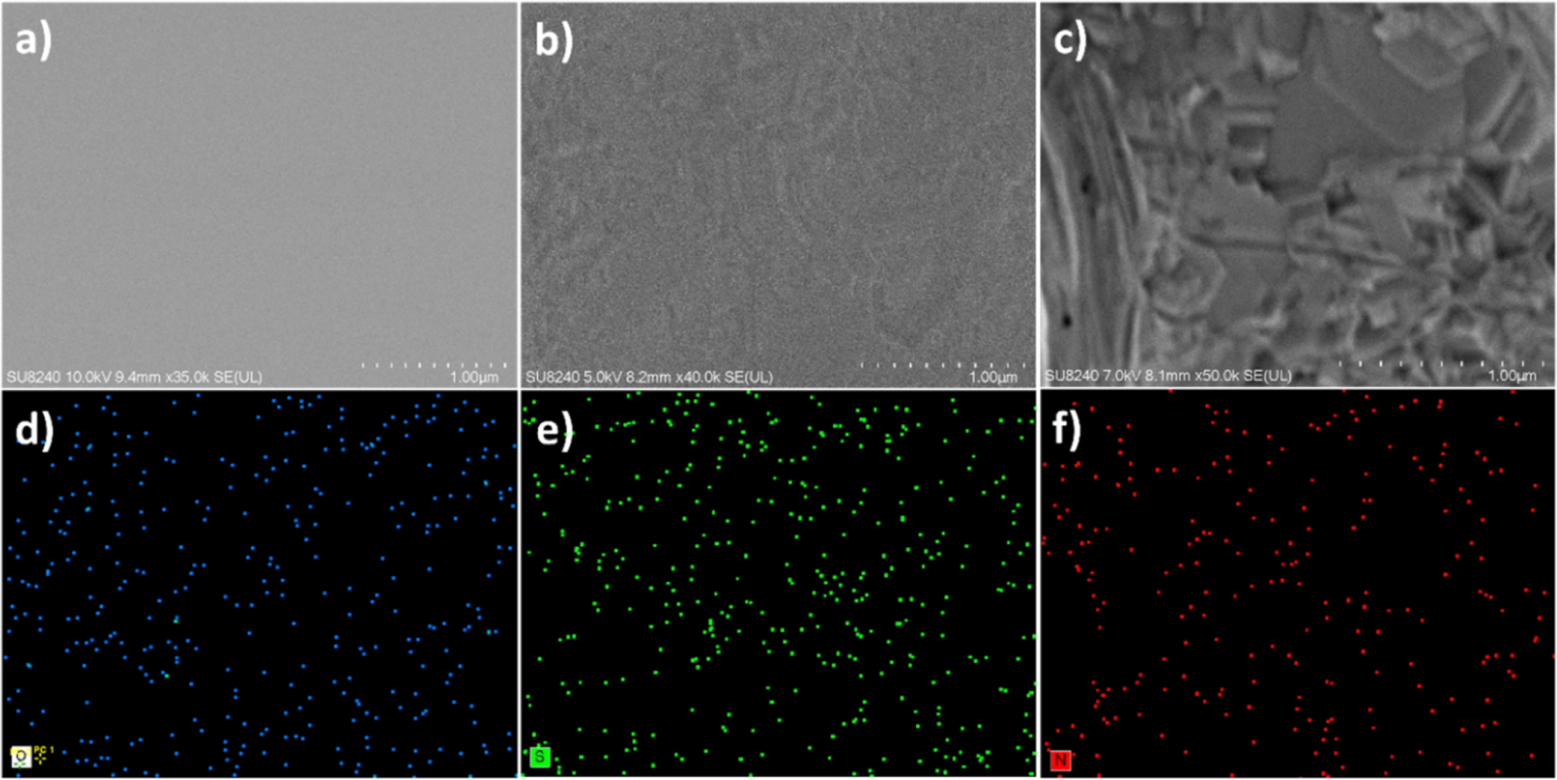
SEM images of the bare Au electrode (a), after polymerization with poly phenol sulfonic (PPS) (c), and adding SPM biomolecule as target (c). EDX characterization after surface modification with PPS leads to the presence of oxygen (d) and sulfuric (e) elements in the spectra and nitrogen group after adding the SPM biomolecule (f).

SEM conducted further exploration of the surface morphology. The SEM micrographs in Figure 3b,c affirm the successful polymerization of MIP nanofilm and its subsequent interaction with SPM, respectively. As illustrated in the EDX images, following polymerization (Figure 3d,e), there was an increase in the presence and density of oxygen and sulfuric groups, indicative of MIP deposition. Subsequently, after interaction with SPM, the emergence of nitrogen groups as a critical element in the protein structure becomes apparent.

XRD was utilized for the examination of structural changes and atomic–level interactions. Adjustments induced by r-GO and the subsequent polymerization procedures have led to observable modifications in the XRD diagrams (Figure S3a). Following r-GO modification, the heightened intensity at 9.8° (002) indicates improved crystallinity, orderly r-GO layers, and potentially new phases emerged as a result of interaction with the Au substrate.^66,67^ In addition, after the polymerization process, a notable enhancement in signal intensity at 9.8° implies a robust interplay between the PPS and r-GO, potentially resulting in a distinct crystalline structure or a more organized arrangement of the PPS-r-GO amalgam on the Au substrate.^68,69^ The emergence of new peaks at 44.8 and 64.5° may be ascribed to specific crystalline structures of the polymers or their interaction with r-GO, denoting the incorporation of novel structural characteristics or compounds during the polymerization phase^68^ Moreover, the heightened magnitude of the peak observed at 38.5° (111) may be associated with the favored alignment of nanocrystallites along the (111) axis as each layer developed.^70^ Additionally, the presence of a peak at 42° may be attributed to the turbostratic structure of the amorphous carbon substance.^71^ Upon the application of surface modification using SPM, the designated molecule for the PPS, XRD examination indicates a notable reduction in the intensity of the majority of the peaks. This observation implies a plausible interaction between the SPM and the imprinted sites of the PPS nanofilm. It is worth noting that a considerable rise in the intensity at 78.32° suggested a heightened level of crystalline organization or a potential phase change. This could be attributed to the rearrangement of the polymer chains surrounding the SPM molecules. Moreover, the appearance of new peaks at 32.04 and 41.48° validated alterations in the structure caused by the attachment of SPM, possibly indicating interconnected network formation within the PPS nanofilm.^72−75^

The FTIR spectra of PPS imprinted polymer (MIP) and NIP reveal distinct differences. As shown in Figure S3b, both MIP and NIP display characteristic peaks of phenol sulfonic acid, with aromatic ring vibrations at 1400–1600 cm^–1^ and a pronounced peak around 1220 cm^–1^ due to the stretching vibration of aromatic ether chains.^76^ This peak is more pronounced in the MIP spectrum, indicating enhanced chain reproduction during the electropolymerization of phenol sulfonic acid. Additionally, the spectra show a peak around 904 cm^–1^ corresponding to C=C stretching, and a peak at 2863 cm^–1^ related to C–H vibrations.^77−79^

The peak at 1370 cm^–1^ is attributed to the stretching vibrations of the SO_3_ moiety in the electropolymerized nanofilm structure, while the peaks at 3200–3500 cm^–1^ are assigned to the –OH stretching vibrations of phenol.^80,81^ After SPM was introduced, notable changes were observed in the FTIR spectra (Figure S3b). The intensity of the peak at 2358 cm^–1^, associated with –COOH, significantly decreased, and the C=O stretching bond at 1738 cm^–1^ disappeared. New peaks appeared at 1538 cm^–1^ and 1990–2100 cm^–1^, corresponding to N=O and C=C stretching bonds, respectively. Additionally, distinct peaks were observed at 1150–1170 cm^–1^ and 1230–1304 cm^–1^, corresponding to the stretching of the S=O and C=N bonds, respectively. These spectral changes indicate successful binding interactions between SPM and the PPS nanofilm.^82−84^ These results are consistent with prior research that has shown the influence of different alterations and polymerization on materials based on graphene, emphasizing the detailed alteration in the crystalline structure, alignment, and interplays at the nanolevel.

### Spermine Detection Electrochemical Assay

**Figure 4:**
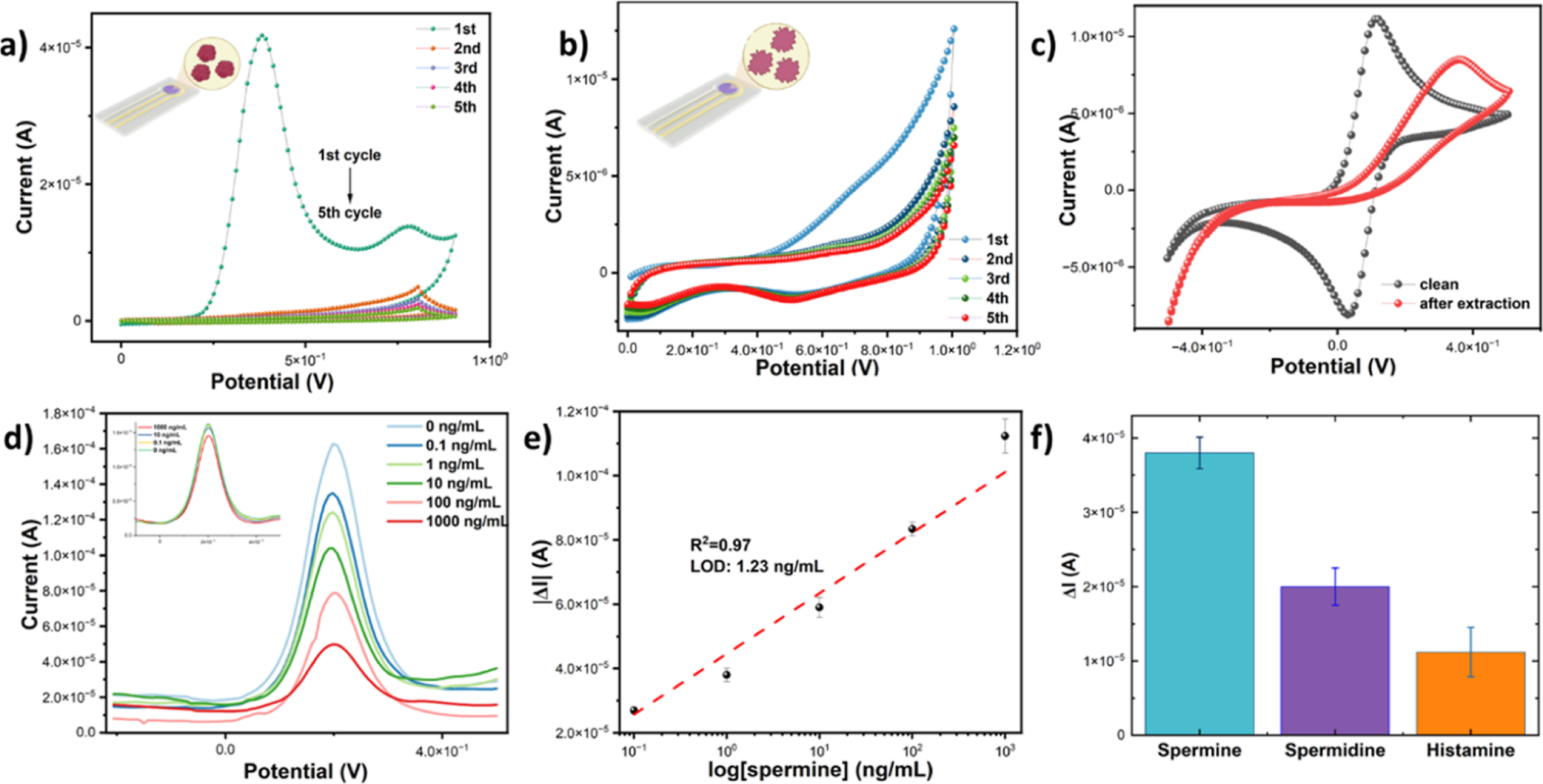
CV for electropolymerization of the nanofilm MIP (an aqueous solution of the P-phenol sulfonic acid, the SPM, and phenol as a cross-linker) on the r-GO/Au electrode (a) and extraction of template in 0.1 M H_2_SO_4_ in the potential range from 0 to 1.05 V at 50 mVs^–1^ scan rate (b). CV after extraction in comparison with a bare Au electrode in the readout solution (−0.004 V vs Ag/AgCl) (c). DPV plot of PPS and NIP (offset) nanofilms when different levels of SPM are added (d) and calibration curve (e) for 0.1–1000 ng/mL level of SPM. Selectivity test of PPS nanofilm showing its responses against the SPM, spermidine, and histamine proteins at a concentration of 1 ng/mL in readout solution (f).

The electrochemical characteristics of both the 3D SPM-PPS nanofilm and NIP were subjected to a comprehensive evaluation through CV, employing the readout solution as the redox probe. As illustrated in Figure 4a, a pronounced reduction in the current intensity of the oxidation peak is observed over consecutive scan cycles, indicative of the gradual formation of a PPS nanofilm that effectively occluded the electrode surface.

This phenomenon imparted a discernible suppression of the voltammetric response, substantiating the inference that the evolving PPS nanofilm hampered the facile electron-transfer process at the electrode–electrolyte interface. Also, the redox peaks disappear after nanofilm electrodeposition, indicating the formation of a PPS nanofilm. The current imprinting method permits the creation of molecularly imprinted sites at or near the surface of the PPS nanofilm. Therefore, it is vital to control the deposition of the PPS nanofilm to achieve an appropriate film thickness and to prevent irreversible entrapment of SPM rendering them unremovable during the washing-out process. This can be achieved by regulating the number of CV cycles and the PPS concentration. Furthermore, optimizing the thickness of the MIP layer serves another purpose: facilitating an adequate charge transfer through the imprinted sites to the electrode. This optimization is crucial for the subsequent detection of SPM rebinding events. Following the removal of the template, a distinctive outcome emerged whereby imprinted cavities were formed, subsequently reinstating the redox signal (Figure 4b). After host–guest recombination with SPM, the reoccupation of these imprinted sites would result in a decrease in the accessibility of the redox markers in the readout solution to the electrode surface leading to an observable decline in the redox signal.

As shown in Figure 4c, the characteristic voltammograms of the redox couple were successfully acquired, illuminating discernible differences between the 3D PPS nanofilm and a bare electrode. Evidently, the redox peak currents exhibited a notable augmentation in the 3D PPS nanofilm. Noteworthy, the modified electrode displayed a diminished peak potential difference (Δ*E*_p_) of 80 mV in contrast to the bare electrode. This trend strongly implies an enhancement in electron-transfer kinetics and a heightened level of reversibility within the redox couple, which are attributable to the intrinsic conductivity of the r-GO/Au.

### Artificial Urinary Spermine Electrochemical Detection via Differential Pulse Voltammetry

The DPV responses, following the interaction between SPM and PPS/NIP nanofilms, serve to demonstrate the performance of the SPM-PPS nanofilm across a range of target concentrations in artificial urine from 0.1 to 1000 ng/mL (Figure 4d). The calibration curve in Figure 4e was generated using the |Δ*I*| values (*I*_2_ (before exposing to SPM)—*I*_1_ (after exposing to SPM)), showing a correlation coefficient (*R*^2^) of 0.97. The LOD for SPM is 1.23 ng/mL (*N* = 3).

The selectivity of the PPS nanofilm for SPM was assessed by its capability to differentiate between SPM and its competitive polyamines, spermidine, and histamine. Spermidine and histamine were chosen because of their structural resemblance to SPM. As illustrated in Figures 4f and S4, the selectivity of the PPS-based sensing platform is confirmed by its specific responses toward SPM but not to other interferences.

### Urinary Spermine Detection with SPM-PPS Nanofilm EGFET Sensing Platform

The SPM-PPS nanofilm functions as the sensing element in an EGFET setup and is divided into two principal units. This design leverages the selectivity of the PPS nanofilm, which is engineered to recognize and bind the SPM specifically. When integrated with the EGFET setup, changes in the electrical properties of the nanofilm upon binding to SPM can be transduced into measurable signals. This setup is designed to offer high sensitivity and selectivity toward SPM, making it an efficient tool for its detection and quantification in complex sample matrices. In this EGFET configuration, the gate terminal of the conventional n-MOSFET serves as the transducer connected to the molecular recognition part of the sensor.

The cross-linker present in the PPS polymer facilitates the polymerization process, leading to the creation of SPM-specific binding sites within the PPS matrix after extraction of the SPM templates. By applying a positive bias to the floating gate electrode (reference electrode), positively charged SPM molecules at pH 7 are drawn to the deprotonated PPS nanofilm on the surface of the sensing platform and bind to the molecularly imprinted sites. The binding and interaction of SPM with the PPS nanofilm induces changes in the surface potential owing to the charge accumulation in the PPS recognition layer.^85^

To evaluate the efficacy of the SPM-PPS nanofilm EGFET for the detection of SPM, a systematic experimental investigation was conducted. This assessment involved the measurement of PPS nanofilm’s responses to various concentrations of SPM in an artificial urine solution. The semiconductor parameter analysis (K2450) was adopted for this NIP and SPM-MIP nanofilm EGFET characterization. The operational mechanism and responsivity of the SPM-PPS nanofilm EGFET were further elucidated through the analysis of the *V*_GS_–*I*_D_ (gate voltage vs drain current) electrical characteristics. This analysis delineates the underlying mechanism governing the performance of the sensing platform. Our hypothesis is that the positively charged target analyte (SPM) approaches and interacts with the surface of the PPS nanofilm. This interaction or binding event prompts a modulation in the surface potential (ψ) of the electrode. Crucially, this modulation in surface potential is directly correlated with alterations in the threshold voltage (*V*_T_) at the interface between the electrolyte and the SPM-PPS nanofilm. Consequently, variations in the concentration of SPM (C) leads to corresponding changes in *V*_T_, which can be mathematically represented by the following relationship (eq 1)^86^
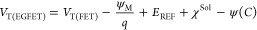
1In this context, the *V*_T_ of the MOSFET is denoted as *V*_T(FET)_, representing the gate threshold voltage for each individual MOSFET. The surface potential of the metal gate is expressed as ψ_M_, while the potential of the reference electrode is defined by *E*_REF_. Additionally, the surface dipole potential of the buffer solution is represented by χ^Sol^. This equation underscores the principle that the sensing mechanism is predicated on the detection of changes in the electrical properties (specifically, *V*_T_) at the electrolyte, SPM-PPS nanofilm interface, triggered by the specific binding of SPM molecules to their templated sites on the PPS nanofilm.^87^ This modality enables the quantification of SPM based on the observed alterations in *V*_T_ (Δ*V*_T_).

**Figure 5:**
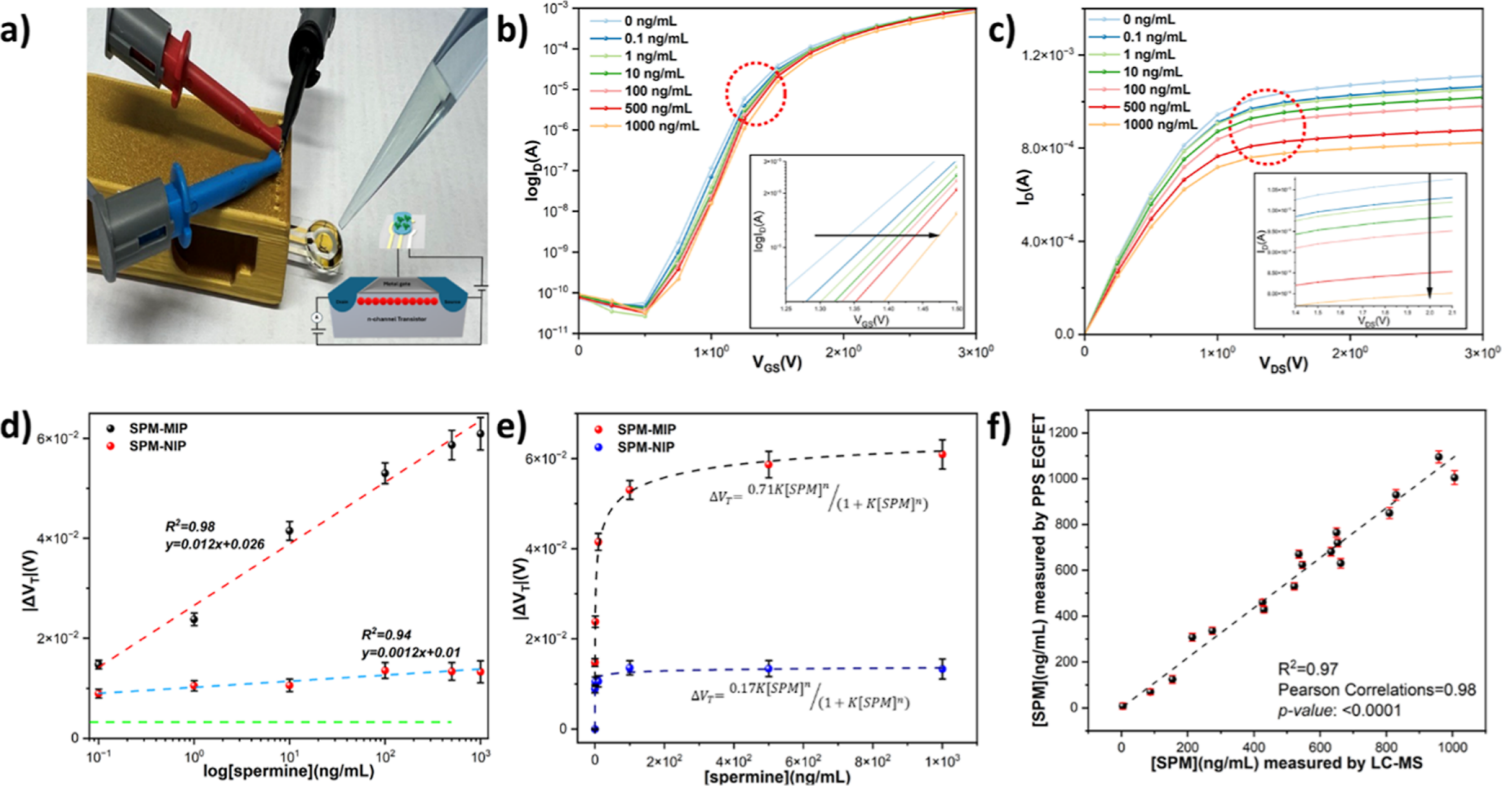
Schematic image of the SPM-MIP nanofilm EGFET (a). Gate voltage-drain current (*I*_D_–*V*_GS_) at *V*_D_ = 2 V (b) and drain voltage-drain current (*V*_DS_–*I*_D_) at *V*_G_ = 3 V (c) characteristics after applying different concentrations of SPM to PPS nanofilm in a readout solution (pH 7.4). The calibration curve for PPS and NIP nanofilm compares the sensitivity and LOD after adding different levels of SPM from 0.1 to 1000 ng/mL in artificial urine (d). The baseline of SPM response, indicated by the green line, is characterized as the mean response augmented by a single standard deviation of three PPS nanofilm EGFETs when subjected to artificial urine in the absence of SPM. The fitted data to a Hill–Langmuir model for both PPS and NIP nanofilms is presented (e). Data from correlative analysis on clinical urine samples revealed a Pearson correlation coefficient of 0.98, demonstrating that PPS nanofilm EGFET perform comparably to liquid chromatography mass spectrometry (LC–MS), the clinical standard technique (f).

Consequently, when a voltage is applied to the extended gate, the surface potential (ψ) changes due to positive SPM binding. This phenomenon leads to a rightward shift in the *V*_GS_–*I*_D_ curves of the n-channel MOSFET (Figure 5b) at constant *V*_D_ = 2 V. It is evidenced from Figure 5b that *V*_T_ elevates as the SPM concentration increases. This is attributable to the increase in carrier density at the SPM-PPS interface, resulting in a higher current flowing through the channel. Also, as illustrated in Figure 5c, the relationship between drain current (*I*_D_) and drain voltage (*V*_D_) can be used to examine the responses of the PPS layer in the presence of SPM. Here, the *I*_D_ at the saturation region can be calculated by
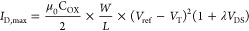
2where electron mobility is depicted by μ_0_ in the channel with the width-to-length ratio of  and length modulation of λ. The capacitance of the unit era is shown as C_OX_, and the voltages applied to the reference electrode and drain are denoted by *V*_ref_ and *V*_T_, respectively. As depicted in Figure 5c, the application of a constant gate voltage (*V*_G_ = 3 V) correlates with a reduction in *I*_D,max_ as the SPM level increases. This decline is the result of the binding interaction between the SPM and the PPS nanofilm, causing a reduction of the effective voltage applied to the gate and a decrease in I_D_.

These results indicate that the gate surface potential of the PPS EGFET responds to the presence of SPM, enabling detection at a concentration of as low as 0.1 ng/mL (Figure 5d). In this context, the performance of the SPM-PPS nanofilm EGFET was examined over a spectrum of target concentrations within artificial urine, ranging from 0.1 to 1000 ng/mL and a calibration curve was constructed utilizing the |Δ*V*_T_| values, yielding an *R*^2^ of 0.98 (Figure 5d). This outcome contrasts with that of the NIP nanofilm EGFET, which shows an *R*^2^ of 0.94 and a sensitivity 10 times lower. Consequently, a notably high imprinting factor (IF ≈ 4.1) is derived from comparing the sensitivities of the MIP and NIP platforms to SPM, underpinning the MIP platform’s enhanced specificity and efficiency.

The Hill–Langmuir model is employed to investigate the binding affinity across a linear detection range of 0.1–1000 ng/mL.^88^ This range corresponds closely to the typical concentration of SPM found in human urine (410.2 ± 136.0 ng/mL).^18,89,90^ Here, Hill–Langmuir isotherms are used to study the adsorption of SPM onto predetermined sites on the PPS and NIP nanofilms. Each site accommodates only one SPM molecule, and the immobilized SPM molecules do not interact with each other. The SPM binding isotherm is adjusted to empirical data to determine the parameters of the isotherm according to eq 3.^88^
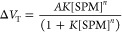
3Here, *A* represents the maximum response when all binding sites are occupied, [SPM] is the concentration of the applied SPM solution, *K* is an effective dissociation constant that describes the SPM concentration producing half occupation of the binding sites, and n is the Hill coefficient, indicating the cooperativity of the binding process.

The dynamic interaction occurring between the SPM and the PPS is visually represented as
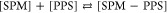
4

Therefore, the value known as the binding constant *K* can be depicted as the relationship between the dissociation and association rate constants, *k*_d_/*k*_a_.
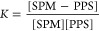
5

The revamped Langmuir isotherm equation can be depicted as
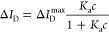
6where, Δ*I*_D_^max^ is the maximum value of the *I*_D_, beyond which further increases in the SPM concentrations do not increase the current.

According to experimental data and parameters obtained from the best-fit analysis of the Hill–Langmuir model (*R*^2^ = 0.99), the comparison between PPS and NIP nanofilm reveals significant differences in their binding characteristics (Figure 5e). PPS exhibits a notably higher capacity (*A* = 0.71) for SPM compared to NIP (*A* = 0.017), indicating its superior ability to bind or absorb the SPM. Additionally, PPS demonstrates a stronger affinity (*K* = 0.55) for the SPM relative to NIP (*K* = 1.33). Both PPS and NIP exhibit negative cooperativity, indicating a decrease in the binding affinity with successive SPM bindings, with NIP (*n* = 0.12) displaying more pronounced negative cooperativity compared to that of MIP (*n* = 0.34). These findings highlight the importance of considering both capacity and affinity characteristics for SPM binding studies, with PPS offering greater efficiency and efficacy in SPM binding compared to NIP.^64,65,88,91−94^

### PPS EGFET Benchmarking by Clinical Samples

The efficacy of the PPS EGFET was rigorously assessed in relation to the established clinical gold standard methodology utilizing liquid chromatography–mass spectrometry (LC–MS). A total of 18 clinical urinary specimens were arbitrarily selected for concurrent analysis employing both the PPS EGFET and LC–MS. Comparative analysis reveals a substantial Pearson correlation coefficient (0.98), with *R*^2^ = 0.97 and absence of a significant difference between the two analytical groups (*p*-value < 0.0001) (Table S2, and Figure 5f). This bench marking from clinical samples substantiates that the PPS EGFET constitutes a promising analytical system, offering a practical instrument for the diagnosis of PCa.

The performance of the PPS nanofilm in SPM detection is compared with those of previous works (Table S3). Few studies have explored the electrochemical detection of spermine. Table S3 presents a comparative analysis of various MIP-based sensing platforms for spermine detection, highlighting the advantages of our PPS sensor. This study demonstrates superior sensitivity (low LOD) and a broad linear detection range. Integrating the PPS with a FET establishes a portable detection system in a groundbreaking manner. Thus, the PPS platform offers a reliable and cost-effective platform for urinary spermine diagnosis and underscores the potential for future advancements in noninvasive PCa detection.

## Materials and Method

### Molecular-Imprinted Polymer Modification

The fabricated Au electrode on a glass substrate was electrochemically cleaned in 0.1 M H_2_SO_4_ via CV, in the potential range from 0 to 1.05 V at a 50 V s^–1^ scan rate for 12 cyclic scans, before depositing the r-GO and the MIP layers. Then, the electrodes were rinsed with DI water and dried under a nitrogen stream. The deposition of the r-GO (0.1 mg/mL) on the electrode surface was conducted by applying −1.2 V on the Au working electrode for 120 s. Then, the modified electrode (r-GO/Au) was electropolymerized in an aqueous solution of the monomer (P-phenol sulfonic acid, 0.2 mM), the template (SPM, 0.1 mM), and cross-linker (Phenol, 1 mM). Before the electropolymerization of the MIP layer, the solution was bubbled with nitrogen for 30 min to remove dissolved oxygen. The electropolymerization was conducted via CV, in the potential range from 0 to 0.9 V at 50 mVs^–1^ for 5 cyclic scans. Specific binding sites on the MIP layer were formed after removing the templates by the extraction procedure in 0.1 M H_2_SO_4_ via CV, in the potential range from 0 to 1.05 V at 50 mVs^–1^ scan rate for 20 cyclic scans.

As illustrated in Figure 1, the 3D P-phenol sulfonic acid MIP nanofilm (PPS nanofilm) has imprinted the size, shape, and specific functional groups of SPM, which results in its high specificity and selectivity toward SPM. Besides, the thickness of the PPS nanofilm can be controlled by the number of CV scans (during the electropolymerization process) that help enhance each sensor’s repeatability.

### Electrochemical Measurement

All electrochemical experiments were performed on an Autolab controlled by NOVA. DPV was used to determine SPM in artificial urine (EN 1616:1999). DVP was recorded in the potential range from −0.5 to 0.5 V at a scan rate of 8 mVs^–1^ and a step potential and modulation amplitude of 50 mV. The current signal was measured at the redox potential of the redox marker (ferrocene-methanol) in the readout solution (vs Ag/AgCl).

### Clinical Sample Preparation

Eighteen urine specimens obtained from the Beatson Cancer Research Institute are divided into five smaller aliquots of 4 mL each and subsequently preserved in a −80 °C cryogenic freezer for prospective utilization. Each specimen is subjected to thawing and pipetting. Urine specimens are conveyed into the tube by utilizing a syringe filter to eliminate the solid phase of the samples, after which they are aliquoted into four sections (1 mL each) and stored in a −20 °C freezer. One aliquot is dispatched to the Chemistry Department to ascertain the SPM concentration through liquid chromatography–mass spectrometry (LC–MS), while the second aliquot is designated for SPM quantification utilizing our PPS EGFET platform.

## Conclusions

This work introduces an innovative and effective approach for spermine (SPM) detection through the employment of DPV and an EGFET integrated with a nanofilm of a MIP. The crucial stages in the advancement of this susceptible and selective sensing platform included the successful construction and analysis of the pseudoreference electrode and the engineered-binding interface.

The Ag/AgCl pseudoreference electrode demonstrated high reliability and reproducibility, with the optimized granular structure to enhance conductivity and reduce interference noises. Electrochemical analysis showed that the PPS nanofilm successfully modified the electrode’s surface, creating imprinted complementary binding sites for SPM. Utilizing DPV, the effectiveness of the system in detecting SPM in artificial urine over a wide range of concentrations (0.1–1000 ng/mL) was demonstrated, showcasing a 1.23 ng/mL LOD, and high selectivity by distinguishing SPM from its competitive proteins, spermidine, and histamine.

The PPS nanofilm integrated with the EGFET setup allows accurate and rapid detection of SPM. Analysis of the electrical properties showed that changes in surface potential, due to SPM binding, led to variations in the threshold voltage of the MOSFET. This validated the system’s sensitivity, with an *R*^2^ of 0.98 and a significantly high imprinting factor (IF ≈ 4.1) when comparing the sensitivities of the MIP and NIP systems to SPM. The use of the Hill–Langmuir model to analyze the binding data highlighted the platform’s impressive binding capacity and affinity for SPM, demonstrating superior performance efficiency compared to NIP. This study introduces a robust, miniaturized, and cost-effective EGFET-based sensing platform for SPM detection, with potential applications in medical diagnostics and monitoring of PCa biomarkers.

## Supporting Information Available

Details on material, substrate preparation, pseudoreference electrode fabrication, electrochemical measurement, electrical setup and analysis, readout solution preparation, CV analysis of pseudoreference electrode, AFM images, table of AFM characteristic parameters, XRD, FTIR characterizations, and comparison to the existing work (PDF)

## Abbreviations

PCa: prostate cancer
MIP: molecular-imprinted polymer
NIP: nonimprinted polymer
PPS: poly phenol sulfonic
SPM: spermine
DPV: differential pulse voltammetry
EGFET: extended field-effect transistor
