# Supplementary material for: Next-Gen Point-of-Care Tool for Ultra-Sensitive Detection of Urinary Spermine for Prostate Cancer Diagnosis
Source: ACS Sens. 2025 Apr 11;10(4):2640–51. doi: 10.1021/acssensors.4c03250 (PMC12038882; doi:10.1021/acssensors.4c03250)
Supplement: Supplementary file 1 — se4c03250_si_001.pdf [file se4c03250_si_001.pdf]

Supplementary information

Next-Gen Point-of-Care Tool for Ultra-Sensitive Detection of Urinary Spermine for Prostate Cancer Diagnosis

Parisa Dehghani*[a], Mostafa Salehirozveh[b], Ataollah Tajabadi[a], Chi Chung Yeung[c], Michael Lam[d], Hing Y. Leung[e], Vellaisamy A. L. Roy*[c]

[a] James Watt School of Engineering, University of Glasgow, Glasgow G12 8QQ, United Kingdom

[b] Department of Physics and Astronomy, University of Bologna, 40126 Bologna, Italy

[c] School of Science and Technology, Hong Kong Metropolitan University, Ho Man Tin, Hong Kong

[d] Department of Chemistry, City University of Hong Kong, Hong Kong

[e] Cancer Research UK Scotland Institute, Glasgow, G61 1BD United Kingdom.; School of Cancer Sciences, MVLS, University of Glasgow, Glasgow G61 1BD, United Kingdom.

*Corresponding authors' E-mail: vroy@hkmu.edu.hk, parisa.dehghani@glasgow.ac.uk

MATERIAL AND METHOD

Material and Apparatus:

The clinical specimens analyzed in this investigation were procured from the NHS Research Scotland Greater Glasgow and Clyde Biorepository (referred to as Glasgow Biorepository thereafter), with the requisite consent and ethical endorsement obtained in accordance with tissue bank REC 22/WS/0020. The reduced-graphene oxide (r-GO), spermine (SPM), spermidine, Histamine, p-phenol sulfonic, phenol, Ferrocene-methanol, Dimethylsulfoxide (DMSO), and glass were purchased from Sigma-Aldrich. Ferric Chloride ($FeCl_3$) 40%, Sulfuric acid (H_2SO_4), and Sodium nitrate ($NaNO_3$) were provided from Merck. The highest grade of reagents is utilised for all commercial orders, eliminating the need for predistillation prior to their use. All the glassware was purchased from Thermo Fisher Scientific. The atomic force microscopy (Bruker, Dimension Icon), scanning electron microscopy (SEM) system and energy-dispersive X-ray (EDX) (Phenom XL Benchtop, Thermo Fisher Scientific in the USA) were utilised for the electrode surfaces' structure and dimensions, along with their chemical properties characterisation. A Heidolph Reax top shaker vortex mixer (Germany) was utilised to uniformly mix polymer and spermine samples. A Nicolet iS5 Fourier transform infrared (FTIR) spectrophotometer with attenuated total reflectance (ATR) accessory was used for

FTIR analysis of the electrode. Agilent 6546 Q-TOF-MS High Resolution Accurate Mass Spectrometer (LC-MS) was used for quantification of spermine.

Methodology:

Substrate preparation:
A cover slip was selected to be used as a substrate. An established cleaning procedure was applied to eliminate the contamination on the surface of the substrate [1, 2]. Afterwards, an electron beam was employed to deposit a layer of Au onto the surface of the glass substrate. To enhance the adhesion between the Au layer with a thickness of 100 nm and the glass, the 20 nm Ti was utilised as an adhesive layer.

Pseudo-reference electrode fabrication

To construct the pseudo-reference electrode, 200 nm silver was deposited by an electron beam in conjunction with a shadow mask for precise fabrication. After drying the deposited silver electrodes, they were subjected to a chlorination process using $FeCl_3$. Chlorination was conducted for 60 seconds with varying concentrations of $FeCl_3$, where the average estimated thickness of the AgCl layer was around 123 nm [3]. The morphological characterisation of the fabricated pseudo-reference electrode was studied using an SEM coupled with an EDX analysis system. The electrochemical performance of the electrode was assessed using a potentiostat/galvanostat Autolab.

Electrochemical measurement:

All electrochemical experiments were performed on an Autolab controlled by NOVA. Differential pulse voltammetry (DPV) was used to determine SPM in artificial urine (EN 1616:1999). DVP was recorded in the potential range from −0.5 to 0.5 V, at a scan rate of 8 mVs^{-1} and a step potential and modulation amplitude of 50 mV. The current signal was measured at the redox potential of the redox marker (Ferrocene-methanol) in the readout solution (vs Ag/AgCl).

Electrical setup and analysis

Various concentrations of SPM ranging from 0.1-1000 ng/mL are prepared in artificial urine at pH 7.4. To examine the PPS-MIP EFGET response to different SPM concentrations, 100 µL of the sample is applied to the surface of a modified electrode for 10 min followed by a rinse with DI water and gentle drying with N_2 gas. This electrode is pre-incubated in a readout solution for 30 minutes to stabilise. Subsequently, the measurement is done in a readout solution (pH 7.4). The gate voltage (V_G) is varied from 0 to 3 V, while a constant voltage of 2 V is maintained between the source and drain contacts to ensure the MOSFET operates in the linear regime. The I_{DS}–V_{GS} characteristics of the n-channel MOSFET are documented as the response of the EGFET sensor to various SPM concentrations. Additionally, V_G is held at a steady 3 V, and the drain voltage (V_D) is varied from 0 to 3 V to examine the I_{DS}–V_{DS} characteristics of the sensor.

Readout solution preparation:

To prepare the readout solution 1mM Ferrocene-methanol was dissolved in 1% dimethylsulfoxide (DMSO), and then, 0.1 M $NaNO_3$ was added to the solution.

Urine samples preparation

10 urine samples from patients being investigated for prostate cancer within NHS Greater Glasgow and Clyde were obtained via the Glasgow Biorepository and were stored in a -80 ˚C freezer as 4 mL aliquots until required for analysis. Each sample was thawed and pipetted before being transferred to a 10 mL centrifuge tube. Urine samples were transferred to the tube via a syringe filter to filter out the solid phase of samples. The samples are centrifuged at 3000 rpm for 5 min, then aliquoted to 4 sections (1 mL) and stored in a -20 ˚C freezer.

RESULTS AND DISCUSSION

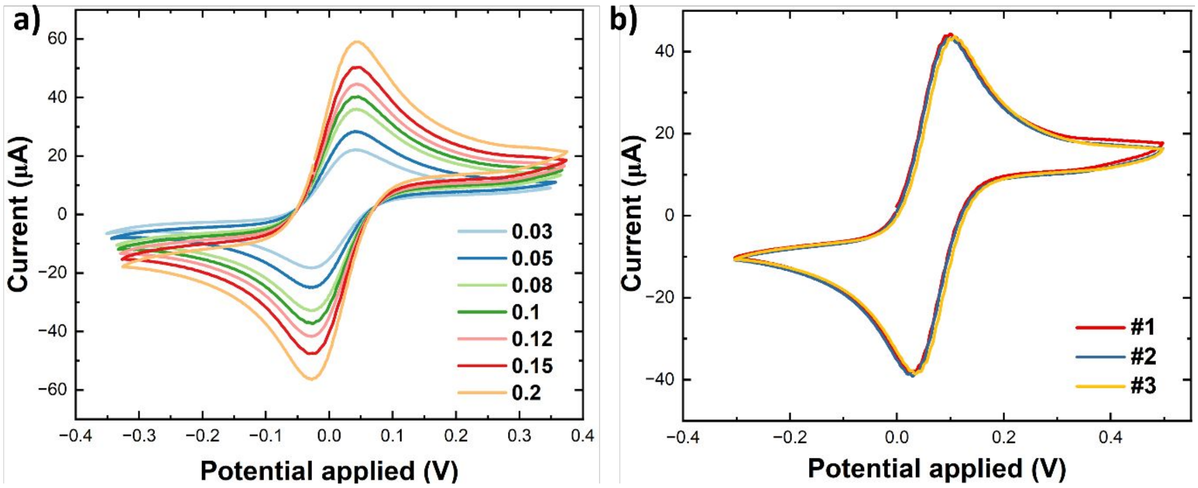

Figure S1. Cyclic- Voltammetry (CV) in readout solution to study the performance of pseudo-reference electrode at different scan rates (a), and its reliability by using three different fabricated pseudo-reference electrodes.

Development of the Binding Surface:

In the design of our molecularly imprinted polymer (MIP) for spermine (SPM) detection P-phenol sulfonic acid as the primary functional monomer, phenol as a cross-linker, and SPM as the template were employed. The phenolic and sulfonic acid groups in P-phenol sulfonic acid enable robust non-covalent interactions such as hydrogen bonding, electrostatic interactions, and π-π stacking with the multiple amine groups of SPM. During polymerization, these interactions orient and stabilize SPM within the growing polymer matrix, ensuring that the resultant imprinted cavities closely match SPM's size, conformation, and functional groups. Phenol cross-linking further enhances the rigidity and stability of the polymer network, mitigating cavity collapse once the template is removed.

Following polymerization, SPM is extracted, leaving behind highly specific recognition sites that selectively rebind SPM upon exposure to a sample. This selective recognition relies on the

same non-covalent interactions that guided the initial template–monomer complex formation, and can be transduced electrochemically or optically, depending on the chosen sensor design. The phenol-cross-linked polymer matrix provides both mechanical and chemical stability, thereby improving detection reproducibility and reducing sensor degradation over time. Such high specificity and robustness are especially valuable for detecting clinically relevant SPM levels associated with PCa.

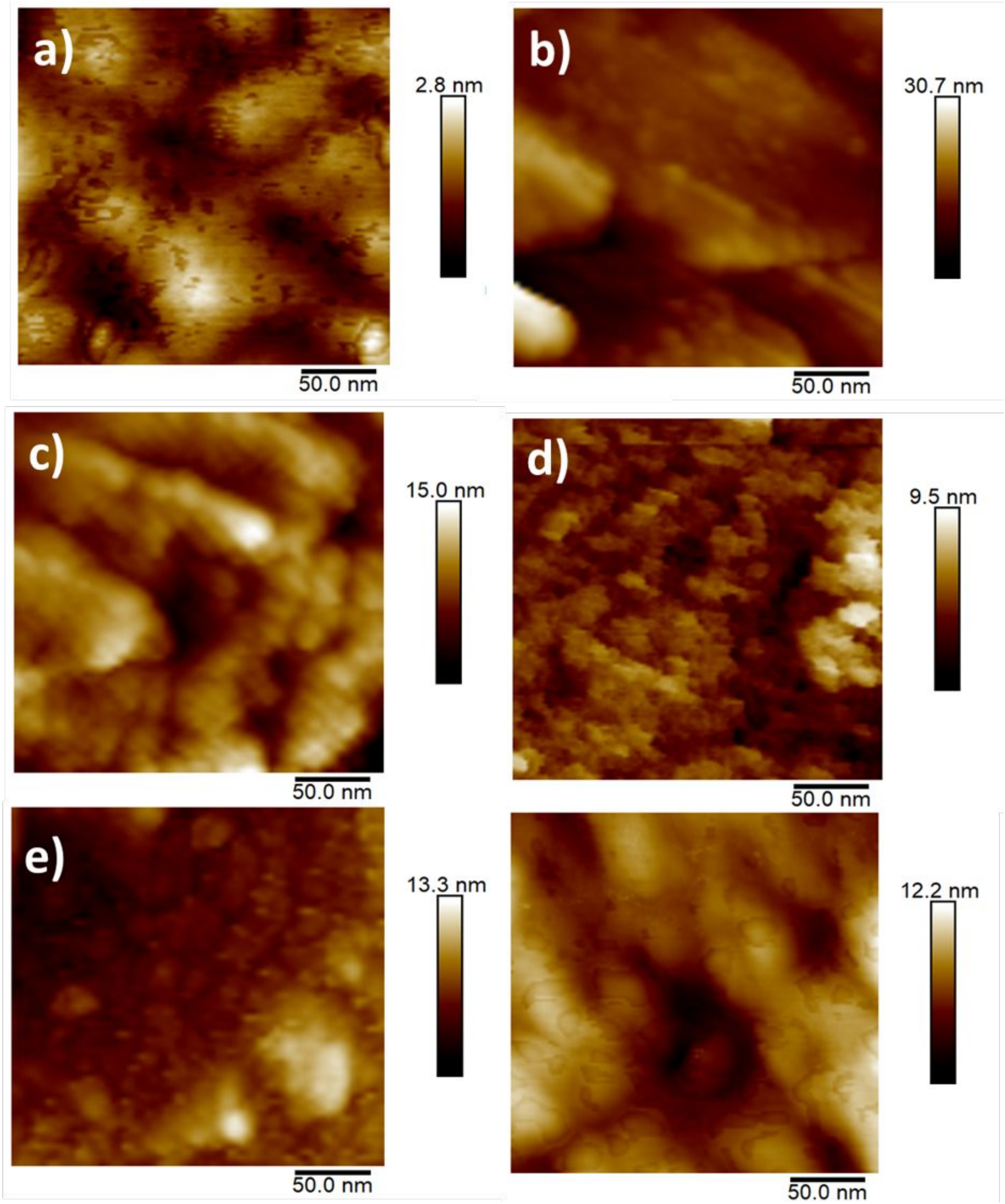

Figure S2. The AFM images from the Au electrode before (a) and after (b) the electropolymerization of PPS nanofilm. Also, surface analysis is conducted on the modified electrode after extraction (c) and intr4oducing the SPM (d) too. The topography of NIP-nanofilm is after the polymerisation (e) and extraction steps are presented.

Table S1. AFM characteristic parameters related to the PPS and NIP nanofilm.

Sample	Thickness (nm)	Roughness (nm)
**Bare**	2.8	0.7
**PPS (MIP) (before extraction)**	30.7	3.4
**PPS (MIP) (after extraction)**	15	5.17
**SPM-PPS (MIP)**	9.5	1.2
**NIP (before extraction)**	12.2	1.91
**NIP (after extraction)**	13.3	2.13

As depicted in the AFM images (Figures S2a–S2f), the surface morphology of the electrodes changed substantially during the different stages of MIP nanofilm formation, template extraction, and rebinding. Initially, the thickness and root mean square (RMS) roughness of the substrate increased after deposition of the MIP nanofilm (Figure S2b), indicating the successful coating of the polymer layer on the underlying electrode. The AFM height profiles confirm that the polymer uniformly covered the surface, forming a continuous film. Following template extraction, a more pronounced enhancement in RMS roughness was observed (Figure S2c). This notable increase suggests that the removal of spermine (SPM) from the polymeric scaffold left behind molecularly imprinted cavities, effectively creating a more uneven topography. The formation of these cavities is a hallmark of MIP technology, wherein the extracted template regions serve as specific binding sites for the target analyte. Upon reintroduction of SPM, the roughness partially decreased, reflecting the occupation of the previously formed binding sites within the imprinted film (Figure S2d). This resurgence in surface coverage demonstrates the strong affinity between the imprinted cavities and the target spermine molecules. The decrease in RMS roughness, compared to the extracted state, further confirms that the recognition sites are selective and capable of being refilled, consistent with the principles of molecular imprinting. In contrast, the non-imprinted polymer (NIP) control showed minimal change in thickness or roughness before and after the same extraction treatment (Figures S2e and S2f). Because the NIP does not contain cavities tailored to spermine, its surface morphology remains relatively invariant, underscoring the specificity of the MIP architecture. A summary of the thickness and RMS roughness for both the MIP and NIP at each step of the process is provided in Table S1.

Taken together, these AFM observations highlight the successful fabrication of a selective MIP nanofilm for spermine detection. The shifts in surface roughness during template removal and subsequent rebinding provide a clear, visual confirmation of the imprinting process. Moreover, the stable morphology of the NIP under comparable conditions underscores the selective nature of the imprinted sites and validates that the observed changes are indeed attributable to specific interactions between the MIP and spermine.

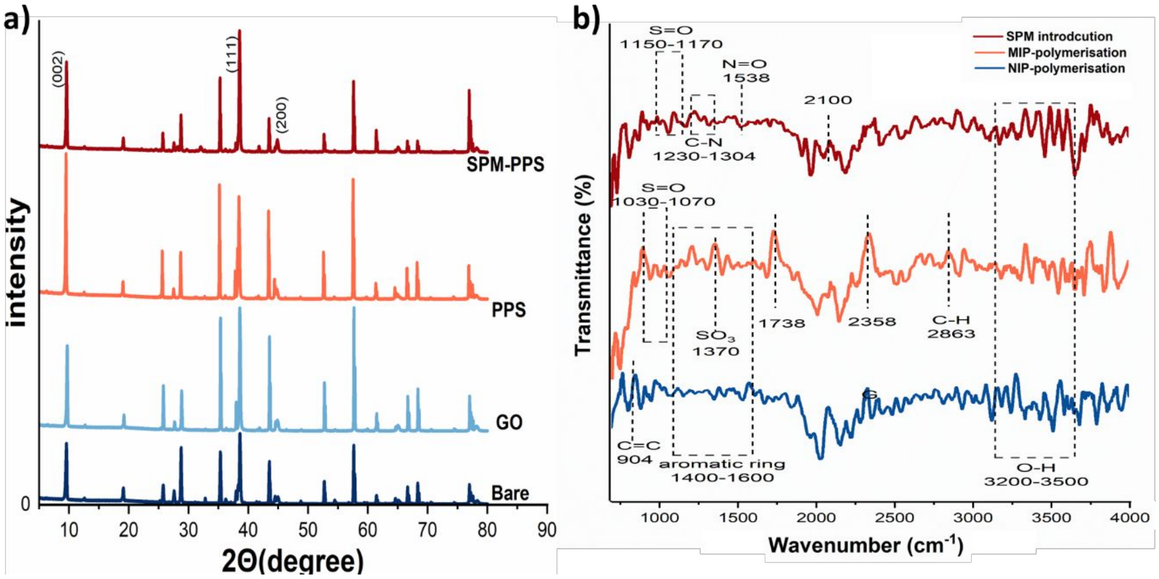

Figure 3. a) XRD image of the bare (Au) electrode, after modification with Go, PPS-MIP, and introduction of SPM biomolecule. b) FTIR spectrum of PPS and NIP nanofilms and after the introduction of SPM.

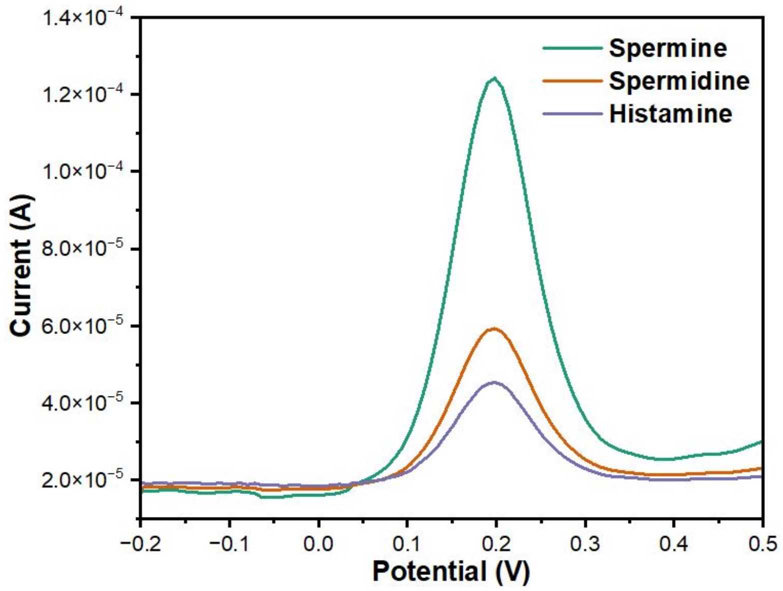

Figure S4 The selectivity test of poly phenol sulfonic (PPS) nanofilm when different competitive analytes including spermidine and Histamine are added.

Table S2. Correlative analysis data of measurements obtained from clinical samples and PPS EGFET

#Sample	[SPM](ng/mL) measured by ICP-MS	[SPM](ng/mL) measured by PPS EGFET
**1**	4.31363	7.86±14
**2**	87.37213	70.1±12.98
**3**	154.19647	124.6±17.1
**4**	214.4061	307.12±17.09
**5**	274	335.57±16.24
**6**	426.01815	460.09±13.21
**7**	430.06103	428.38±14.01
**8**	521.84697	530.12±15.32
**9**	535.45772	670.63±17.65

**10**	546	623.62±15.32
**11**	633.02608	681.12±18.61
**12**	649.26076	765.32±19.94
**13**	652.98779	720±16.48
**14**	661.95792	630.44±20.56
**15**	809.45976	850.48±24.24
**16**	829.48463	929.25±23.21
**17**	958.47767	1095.32±25.89
**18**	1006.04465	1005.01±30.14

Table 3 Comparison study of MIP-based sensor for spermine detection.

Target	Electrode	Polymer-MIP	source	Technique	LOD (ng/mL)	Range of detection (ng/mL)	Ref.
**Spermine**	SPE	methacrylic acid	saliva	CV	7×10^{-3}	0-202.34	[4]
**Spermine**	rGO-Cu−CNF	phenol	Artificial serum	DPV	60.72	10^{4}-3×10^{5}	[5]
**Spermine**	Cu−CNF	poly(β-CD)	blood	DPV	0.991	0.5−2×10^{3} 2×10^{3}−10^{5}	[6]
**This work**	Au	phenol	Artificial/real urine	DPV EG-FET	1.23	0.1-1×10^{3}	This work

REFERENCES

(1) Birch, W. Cleaning glass surfaces. In *Sol-Gel Technologies for Glass Producers and Users*, Springer, 2004; pp 19-34.

(2) Cras, J. J.; Rowe-Taitt, C. A.; Nivens, D. A.; Ligler, F. S. Comparison of chemical cleaning methods of glass in preparation for silanization. *Biosensors and bioelectronics* **1999**, *14* (8-9), 683-688.

(3) Dunare, C.; Marland, J.; Blair, E.; Tsiamis, A.; Moorel, F.; Terry, J.; Walton, A.; Smith, S. Test structures for characterising the silver chlorination process during integrated Ag/AgCl reference electrode fabrication. In *2019 IEEE 32nd International Conference on Microelectronic Test Structures (ICMTS)*, 2019; IEEE: pp 58-63.

(4) Athar, S.; Zaman, I.; Liaqat, A.; Afzal, A. Disposable Saliva Sensors for Early Cancer Diagnosis Using Hyper-Cross-Linked Molecularly Imprinted Polymeric Electrocatalysts. *ACS Applied Polymer Materials* **2023**, *5* (12), 10438-10445.

(5) Ali, H.; Verma, N. A Cu–CNF–rGO-functionalized carbon film indicated as a versatile electrode for sensing of biomarkers using electropolymerized recognition elements. *Journal of Materials Science* **2022**, *57* (11), 6345-6360.

(6) Shrivastava, S.; Bairagi, P. K.; Verma, N. Spermine biomarker of cancerous cells voltammetrically detected on a poly (β-cyclodextrin)-electropolymerized carbon film dispersed with Cu-CNFs. *Sensors and Actuators B: Chemical* **2020**, *313*, 128055.
